# Gold nanorod-based multifunctional nanocarrier for synergistic chemo-photothermal therapy in tumors†

**DOI:** 10.1039/c8ra06176a

**Published:** 2018-12-11

**Authors:** Yi Wang, Qiyao Cui, Xiaoshuang Zhao, Tang Qin, Wenjing Wang, Hongmei Sun, Hongda Zhu, Huiling Guo, Honghao Sun

**Affiliations:** School of Bioengineering and Food, Key Laboratory of Fermentation Engineering (Ministry of Education), Key Laboratory of Industrial Microbiology in Hubei, National “111” Center for Cellular Regulation and Molecular Pharmaceutics, Hubei University of Technology Wuhan 430068 China 1848923282@qq.com

## Abstract

Synergistic photothermal therapy (PTT) and chemotherapy is an efficient strategy for tumor therapy. However, it is still a challenge to design a smart delivery system able to release a drug at the appropriate time and site of action. Here, we have synthesized photosensitive molecule 7-(double dodecylamine)-4-hydroxymethylcoumarin which was introduced in a nanocarrier GNR@SiO_2_-DOX@CouC_12_-HA (GSDCH) to achieve manually controlled drug release. The specific nanocarrier was fabricated using a GNR core for photothermal therapy, a mesoporous silica shell for drug loading, and the coumarin moiety as a blocking agent and intelligent controlled switch. In addition, cellular uptake of GSDCH by HeLa cells can be achieved effectively with the help of hyaluronic acid (HA). Owing to the controlled and targeted drug release properties, the GSDCH with photothermal- and chemo-therapy showed significantly enhanced therapeutic efficiency for HeLa tumor-bearing mice compared to the results of single therapy alone. It indicated that the GSDCH had great potential in tumor therapy with negligible systematic toxicity.

## Abstract 2

Synergistic photothermal therapy (PTT) and chemotherapy is an efficient strategy for tumor therapy.

## Graphical abstract

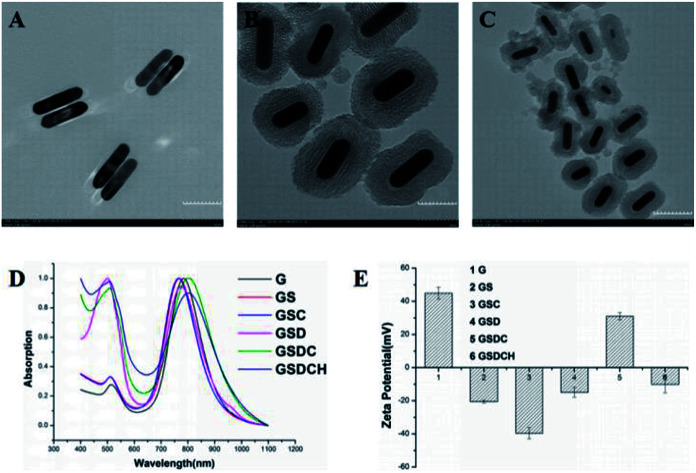

## Introduction

With the development of drug delivery technology, an efficient, convenient controlled-release system is demanded. To precisely control drug release, various stimuli-responsive NPs have been developed, for example, to pH, enzymes, and redox state.^1–3^ These passive controlled release strategies depend on the recognition of subtle environment changes associated with the tumor microenvironment and tumor cells. Nevertheless, it is clear that the tumor microenvironment varies substantially between both patients and tumor types, and even within the same patient or tumor type over time.^4^ Recently, the active controlled release, especially with photo-cleavage trigger, has gained significant attention due to the manually controlled-release of loaded molecules from the carrier systems.^5^ Spatial or temporal controlled release can be achieved through a photo trigger.^6^ The ideal type of light should possess several benefits such as being minimally invasive and simple to perform. Most light-responsive drug delivery systems respond to UV or visible light, such as the molecules with azobenzene moieties, which limits their applications in biomedical field due to the limited penetration depth in tissue.^7^

Among various trigger sources, NIR is the best candidate due to the properties that it can penetrate tissues with sufficient strength and greater spatial accuracy to induce localized hyperthermia.^8–10^ Furthermore, NIR light can be used for photothermal therapy (PTT) in the treatment of cancer.^11,12^ The combination of chemotherapy and hyperthermia could change the fluidity and permeability of the tumor membrane which makes the drug molecule entry the tumor cells and maintain the high drug concentration easily.^13,14^

There are several types of photothermal conversion nanoparticles based on novel metal nanostructures such as gold, silver and palladium, carbon nanomaterials such as graphene oxide, as well as dyes and polymers.^15^ Among various photothermal nanomaterials, gold nanorods (GNRs) is an ideal phototherapeutic agent for PTT because of their tunable longitudinal surface plasmon resonance (LSPR) peak at the first NIR (NIR-I) (650–950 nm) to match NIR resources with different wavelengths.^16–27^ The low drug loading capability and difficult surface modification of naked GNRs limit their further application in targeted drug delivery.^28–30^ Mesoporous silica coating have been successfully applied in targeted drug delivery and tumor therapy due to its uniform and tunable particle/pore sizes, high surface area and pore volume, facile surface functionalization as well as favorable biocompatibility.^31–33^

**Scheme 1:**
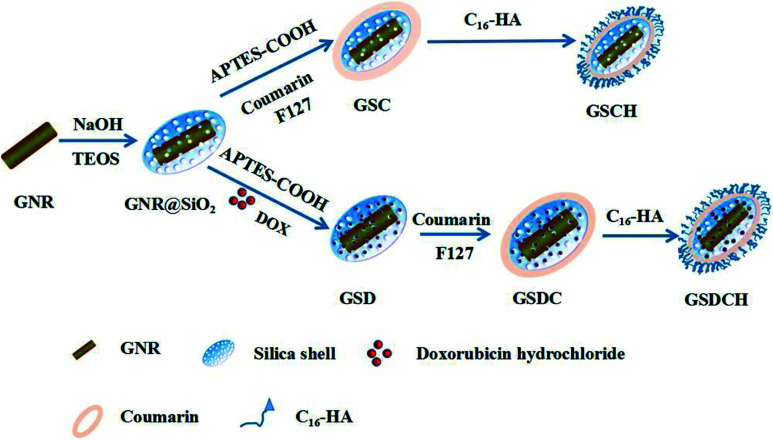
Schematic illustration of the procedure for the fabrication of the multifunctional nanocarrier GSDCH.

Herein, we report a NIR sensitive coumarin derivative coated GNR@SiO_2_ NPs encapsulating DOX by a simple layer by layer coating technique. The coating of coumarin derivative serves as a hydrophobic layer to prevent the drug molecule leakage which results in its ineffective delivery. We designed a novel type of multilayers nanocarrier composed of GNR@SiO_2_-DOX@CouC_12_-HA (GSDCH) for synergistic PTT and chemotherapy. As illustrated in Scheme 1, GNRs were first prepared as a core and then the silica shell was coated to provide drug loading capability. After being functionalized with carboxyl group, DOX was loaded *via* electrostatic interaction. The hydrophobic coumarin moiety was served as blocking agent to prevent DOX leakage before reaching the tumor cells. Then hyaluronic acid (HA) could provide colloid stability and tumor target for this nanocarrier. Under 808 nm NIR laser irradiation, smart light-controlled switch is open and DOX is released. Furthermore, the light absorbance at the plasmon resonance frequency can be converted into heat which results the apoptosis of tumor cells. Therefore, the reported manually controlled-release system represents a potential strategy for synergetic chemo-photothermal therapy.

## Results and discussion

### Preparation and characterization of GSDCH nanocarrier

**Fig. 1:**
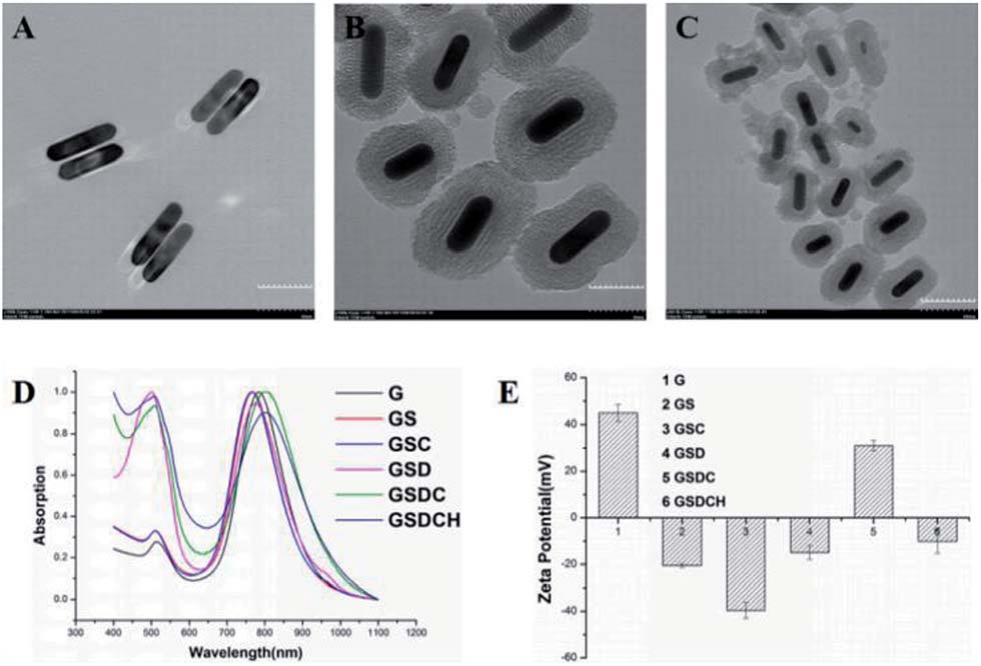
(A) Transmission electron microscope (TEM) image of GNR and (B and C) GNR@SiO_2_. (D) UV-vis-NIR absorbance spectra of GNR (G), GNR@SiO_2_ (GS), GNR@SiO_2_–COOH (GSC), GNR@SiO_2_-DOX (GSD), GNR@SiO_2_-DOX@CouC_12_ (GSDC) and GNR@SiO_2_-DOX@CouC_12_-HA (GSDCH). (E) Zeta potential of GNR (G), GNR@SiO_2_ (GS), GNR@SiO_2_–COOH (GSC), GNR@SiO_2_-DOX (GSD), GNR@SiO_2_-DOX@CouC12 (GSDC) and GNR@SiO_2_-DOX@CouC_12_-HA (GSDCH). The data are represented as a mean ± SD (*n* = 3).

GNRs (Fig. 1A) were synthesized *via* the seed-mediated growth method. The mesoporous structure of GNRs@SiO_2_ (Fig. 1B and C) was clearly observed by the TEM. The thickness of the silica layer was around 15–20 nm. The optical property of GNRs, GNR@SiO_2_, GSC, GSD, GSDC and GSDCH were measured by UV-vis-NIR absorbance spectra (Fig. 1D). According to UV-vis-NIR spectroscopy, the longitudinal surface plasmon resonance (LSPR) peak of GNRs appeared at 785 nm and transverse surface plasmon resonance (TSPR) at 514 nm. After the silica layer was deposited on the as-synthesized rod-shaped GNR, the LSPR was blue-shifted to 768 nm. The result reveals that the GNRs were successfully coated with silica layer. After a series of functionalization, the LSPR peak was finally red-shifted to 802 nm. The UV absorption of pure DOX is around 480–500 nm. When the DOX was loaded, a new shoulder-peaks in the range of 480–500 nm was appeared close TSPR peaks, which suggested that DOX was successfully loaded into the silica layer. The zeta potential results of GNRs, GS, GSC, GSD, GSDC and GSDCH were showed in Fig. 1E. The zeta potential of GNR was +45 ± 3.6 mV, due to the existence of CTAB on the surface of GNRs. After the silica coating, the zeta potential of GS became −20.5 ± 1.0 mV due to the ionization of Si–OH. Subsequently, upon successful conjugation with carboxy group, the zeta potential of GSC was decreased to −39.6 ± 3.5 mV. When loaded with DOX, the zeta potential of GSD was −14.8 ± 3.0 mV, which results from the positive charge of doxorubicin hydrochloride. With the conjugation of coumarin, the zeta potential of GSDC was overturned to +31 ± 2.3 mV due to the existence of abundant ammonium-group of coumarin moiety. After the introduction of C_16_-HA, the zeta potential of GSDCH was converted back into a negative charge (−10.1 ± 5.1 mV). Considering C_16_-HA chain has a negative charge, the result directly proves the successful coating of HA. The 7-(double dodecylamine)-4-hydroxymethylcoumarin was synthesized as a modified reported method (Fig. S1†), and a typical ^1^H NMR spectrum in CDCl_3_ is shown (Fig. S2†).^6^ We examined the stability of GSDCH, the zeta potential of GSDCH was measured for three month (Fig. S3†). The zeta potential results showed no obvious difference in three months (the GSDCH will be ultrasonically treated for three minutes before measured).

DOX content of the nanocarrier was measured and the encapsulation and drug loading rate were investigated. The encapsulation rate and drug loading rate of GSDCH were 97.5% + 0.22% and 22.4% + 2.7%, respectively, indicating that effective drug loading can be achieved through physical adsorption and electrostatic force.
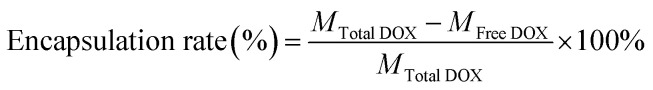

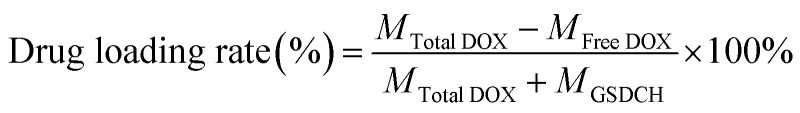


### Photothermal conversion efficiency

**Fig. 2:**
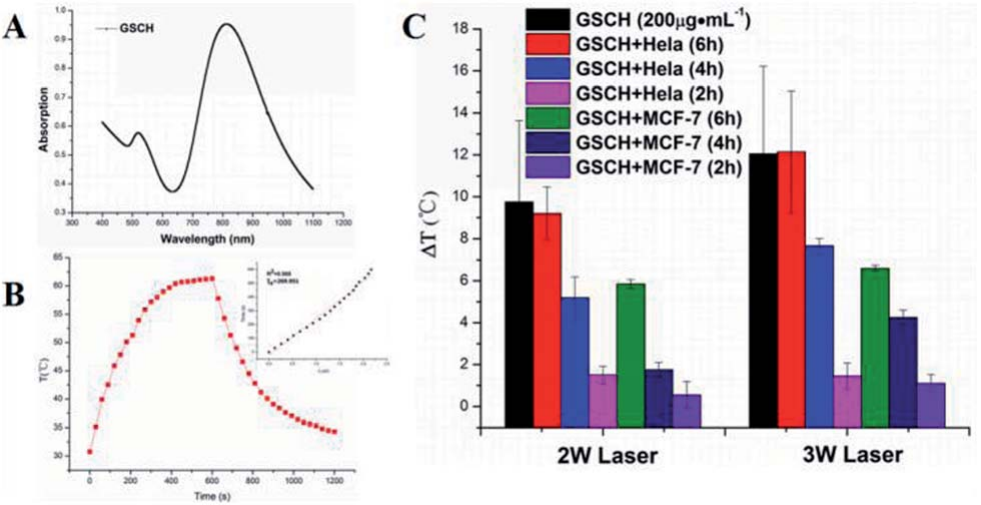
(A) UV-vis-NIR absorption of GSCH (1 mg mL^−1^), *A*_808 nm_ = 0.953. (B) Photothermal effect of the irradiation of the aqueous dispersion of GSCH (1 mg mL^−1^) with the NIR laser (808 nm, 3 W cm^−2^), in which the irradiation lasted to reach the balanceable temperature, and then the laser was shut off. (C) Temperature increase of GSCH (200 μg mL^−1^) incubated with HeLa and MCF-7 cells for different times on 808 nm laser irradiation at 2 W cm^−2^ and 3 W cm^−2^ power density. The data are represented as a mean ± SD (*n* = 3).

To verify the potential of using GSCH in photothermal therapy, GNRs, GS and GSCH solution were exposed to an 808 nm NIR laser at various power densities with water as a control. Since the heating effect of GNR is significantly lower than that of 2 W and 3 W at the power of 1 W (Fig. S10†), the following photothermal experiments are conducted with the power of 2 W and 3 W. Under the NIR irradiation (808 nm, 3 W), the temperature increase of GSCH complex solution (Fig. 2A and B) observably enhanced as the increasing laser power. In obvious contrast to water (7.8 °C increase) (Fig. S8†), the temperature of GSCH solution increased from 30.9 °C to 84.5 °C upon laser irradiation for 10 min. The photothermal efficiency of GSCH was assayed to be ∼28.4%. The result demonstrates that the GSCH have excellent NIR photothermal effect and great potential for using in photothermal therapy. Compared with the GNR (35.4%) (Fig. S4†) and GNR@SiO_2_ (36.8%) (Fig. S5†), GSCH (22.6%) (Fig. S6†) still remains high photothermal efficiency. Under repeated on-off irradiation of 808 nm laser at the irradiation power density of 2 W cm^−2^, the temperature of GNRs aqueous dispersion maintained a coincident reciprocation of the rising and cooling process (Fig. S6†), indicating that the excellent photothermal stability of GNR.

### *In vitro* photothermal assay

The temperature increase of GSCH into HeLa and MCF-7 cells could be examined by digital thermometer (Fig. 2C). Under the irradiation of NIR, the temperature rise increased with the increase of incubation time (Fig. S9†). The temperature increase was depended on the existence of GSCH in cells. The cellular uptake efficiencies for GSCH were significantly increased with increasing the incubation time. After 6 h incubation, the temperature increase of GSCH in HeLa cells was equal to the GSCH solution (200 μg mL^−1^), indicating the maximum cellular uptake of GSCH. The temperature increase of HeLa cells containing GSCH were obviously higher than that of MCF-7 cells because of the over-expression of CD44 receptor on the HeLa membrane. The results demonstrate the GSCH could specifically target HeLa cells.

### Hemolysis

**Fig. 3:**
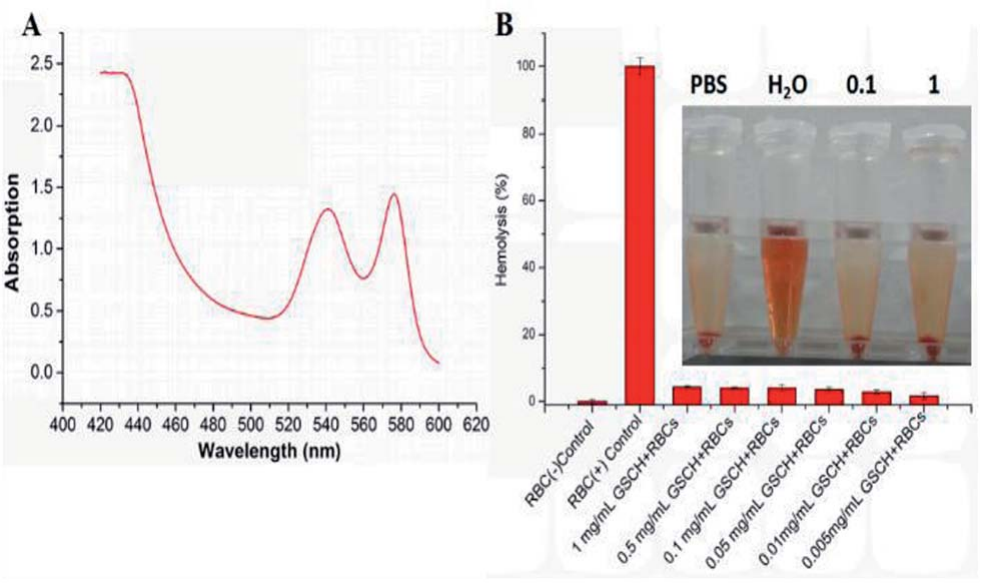
(A) UV-vis-NIR absorption of RBCs in water. (B) Hemolysis assay for GSDCH, using water as a positive control (+) and PBS as a negative control (−). Different concentration of GSDCH were incubated with RBCs for 3 h and the mixtures were centrifuged to detect the presence of hemoglobin by the microplate spectrophotometer at 576 nm. The data are represented as a mean ± SD (*n* = 3).

To evaluate the hemolytic behaviors of GSCH on RBCs, we performed the hemolysis assay by detecting the absorbance of the released hemoglobin from hemolytic RBCs using UV-vis-NIR spectrometer (Fig. 3A). The hemolytic activity of GSCH on RBCs was observed using microplate reader and digital photography (Fig. 3B). It was observed that the GSCH complex was safe on hemolytic front. They caused lysis in 4.5 ± 0.3%, 4.1 ± 0.4%, 4.2 ± 0.9%, 3.6 ± 0.7%, 2.9 ± 0.6% and 1.8 ± 0.9% erythrocytes with different concentration, respectively. Low hemolytic potential of GSCH will bode well for its amicability towards intravenous administration.

### *In vitro* chemo-photothermal therapeutic efficacy of GSDCH nanocarrier

**Fig. 4:**
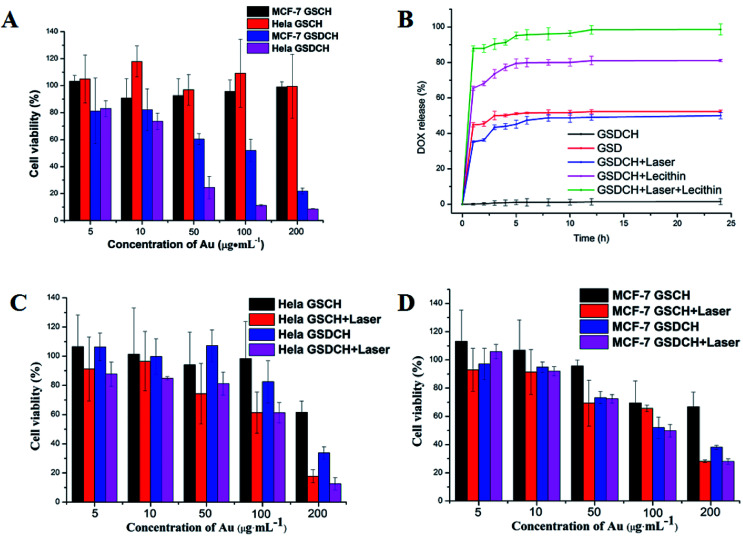
(A) *In vitro* evaluation of HeLa and MCF-7 cell viabilities treated with GSCH and GSDCH after 24 h. (B) Kinetics of laser and lecithin-triggered release. The samples were irradiated with 808 nm NIR laser at the power density of 3 W cm^−2^ for 5 min. *In vitro* evaluation of (C) HeLa and (D) MCF-7 cell viabilities treated with GSCH, GSCH + laser, GSDCH, GSDCH + laser. The samples were irradiated with 808 nm NIR laser at the power density of 3 W cm^−2^ for 30 s. The data are represented as a mean ± SD (*n* = 3).

HeLa (over-expressed CD44 receptor) and MCF-7 cells, acting as the positive and negative group, were used in the MTT assay to detect *in vitro* cytotoxic activity of GSCH and GSDCH. At a high Au concentration (200 μg mL^−1^) of GSCH, more than 80% the cells survival suggests negligible cytotoxicity of GSCH against HeLa and MCF-7 cells (Fig. 4A). However, GSDCH containing DOX are more cytotoxicity to these two cells (cell viability: 21.86% ± 2.26%, 8.66% ± 0.21%, 200 μg mL^−1^). It is supposed that when GSDCH attached to the cytomembrane, the hydrophobic coumarin layer would diffused by the hydrophobic domain of cell membrane, following the release of DOX to kill tumor cells. In order to verify the suggested hydrophobic interaction DOX release mechanism, lecithin was mixed with the GSDCH to mimic the cell membrane interaction process with GSDCH. When adding lecithin, the amount of DOX released was 98.68% (with irradiation) and 81.18% (without irradiation). The results indicated that the hydrophobic lecithin could promote the release of DOX from GSDCH due to the hydrophobic interaction between lecithin and coumarin moiety. Such a irradiation and hydrophobic interaction promoted release behavior of DOX is expected to maximally prevent the drug leakage during circulation and increase the DOX accumulation in tumor cells. To prevent the DOX leaching during the cell membrane cross process, crosslinked or thicker membrane coating of nanocarrier may be applied in the future.

The potential cytotoxicity of GSDCH was evaluated by MTT assay (Fig. 4C and D). Firstly, HeLa and MCF-7 cells were incubated with different concentrations of GSDCH for 24 h. As the concentration of GSDCH increased to 200 μg mL^−1^ (concentration of Au), the cell viabilities of HeLa cell (12.6 ± 4.1%) is lower than MCF-7 cell (28.0 ± 2.0%), respectively.

**Fig. 5:**
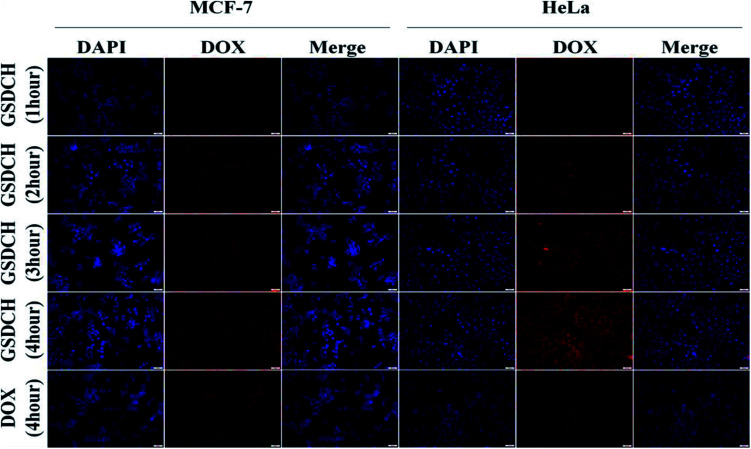
Pictorial evidence of time dependent GSDCH uptake in HeLa and MCF-7 cells. The drug seems to localize completely in nuclear region at 4 hours after GSDCH treatment. However comparative fluorescence from GSDCH and DOX solution treated cells at 4 hours is much lesser than that produced by GSDCH (scale bar, 50 μm).

The result demonstrates that the GSDCH could specifically target HeLa cells. For GSDCH, which is due to the DOX release. Meanwhile, cells incubated with GSCH, the viability rates of cells treatment with irradiation (Fig. 4C and D) are lower than without irradiation (Fig. 4A). The exceeding apoptosis should be caused by PTT. The above results demonstrate the synergistic photothermal and chemotherapy are more efficient than individual therapy alone. Encouraged by results of *in vitro* cytotoxicity and photothermal experiments, we attempted to visually map fate of GSDCH and DOX (Fig. 5). HeLa and MCF-7 cells were cultured in 24 well plates and incubated with GSDCH dispersion (200 μg mL^−1^) equivalent to 25 μg mL^−1^ DOX. After incubated 1, 2, 3 and 4 h with GSDCH, cells were stained with nuclear dye DAPI. As expected, HeLa cells treated with GSDCH exhibit strong red fluorescence (DOX) in the cytoplasm. At 1 and 2 h it was found that slight red fluorescence was observable specifically in cytoplasmic domain only, away from an independently discernible and regularly shaped nuclear region. This suggests successful transmigration of drug across cellular membrane. At 3 and 4 h, intensity of red fluorescence was clearly enhanced. It demonstrates that more DOX were taken up and accumulated in HeLa cells. While both the HeLa and MCF-7 cells were exposed to the same concentration of the nanocarriers, the red fluorescence intensity in HeLa cells was markedly higher than that in MCF-7 cells owing to the overexpressed CD44 receptor on HeLa cells, demonstrating a more efficient cellular uptake of GSDCH active targeting to HeLa cells compared with MCF-7 cells.

**Fig. 6:**
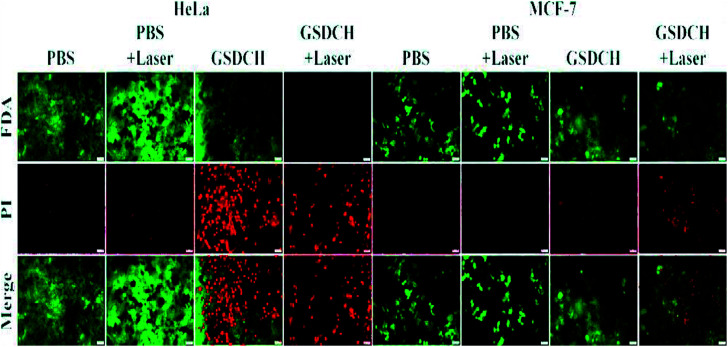
FL images of HeLa and MCF-7 cells after different treatment. Viable cells were stained green with FDA, and dead/later apoptosis cells were floating and eluted, or stained red with PI. The samples were irradiated with 808 nm NIR laser at the power density of 3 W cm^−2^ for 30 s (scale bar, 50 μm).

As shown in Fig. 6, the PBS + laser group displayed no obvious cell death, indicating the slight effect of laser on cells. Compared with HeLa cells treated with GSDCH and GSDCH + laser, HeLa cells treated with GSDCH + laser exhibited almost complete destruction, whereas the cells with GSDCH alone had higher viability efficiency. The above results indicate that the combined effect of the cytotoxicity of DOX and the photothermal effect of GNRs could causing cell damage at greatest degree. Moreover, MCF-7 cells had higher viability efficiencies compared to HeLa cells under the same conditions, further demonstrating the targeting effect of GSDCH.

### *In vivo* thermal imaging

**Fig. 7:**
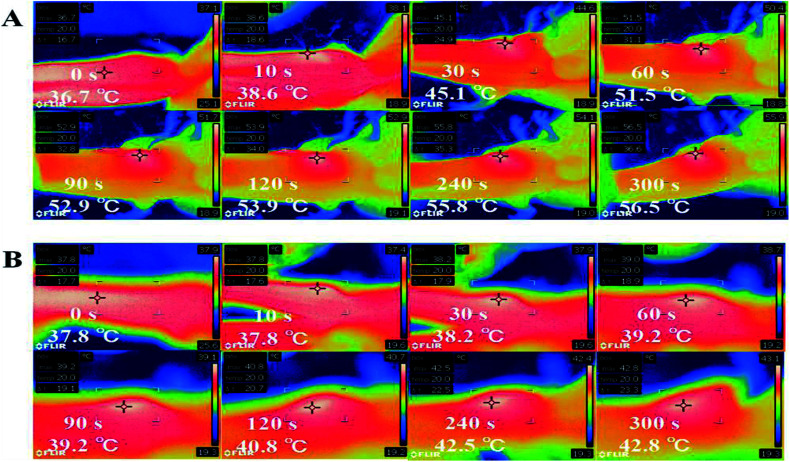
(A) *In vivo* thermal images of tumor-bearing mice after intravenous injection of GSDCH with continuous irradiation of 808 nm laser (2 W cm^−2^) for varied durations. (B) *In vivo* thermal images of tumor-bearing mice after intravenous injection of GSDCH with continuous irradiation of 808 nm laser (2 W cm^−2^) for varied durations.

To monitor *in vivo* photothermal effect generated by GSDCH, a thermal imaging camera was used. After 24 h intravenously injection of GSDCH, the tumor spot of mouse was irradiated with the 808 nm laser for 5 minutes, the temperature of tumor spot increased 20.3 °C (from 36.7 °C to 56.4 °C) (Fig. 7), while the negative group without GSDCH only had 5 °C (37.8 °C to 42.8 °C) increase. These results demonstrate GSDCH have great photothermal effect.

### *In vivo* chemo-photothermal therapy

**Fig. 8:**
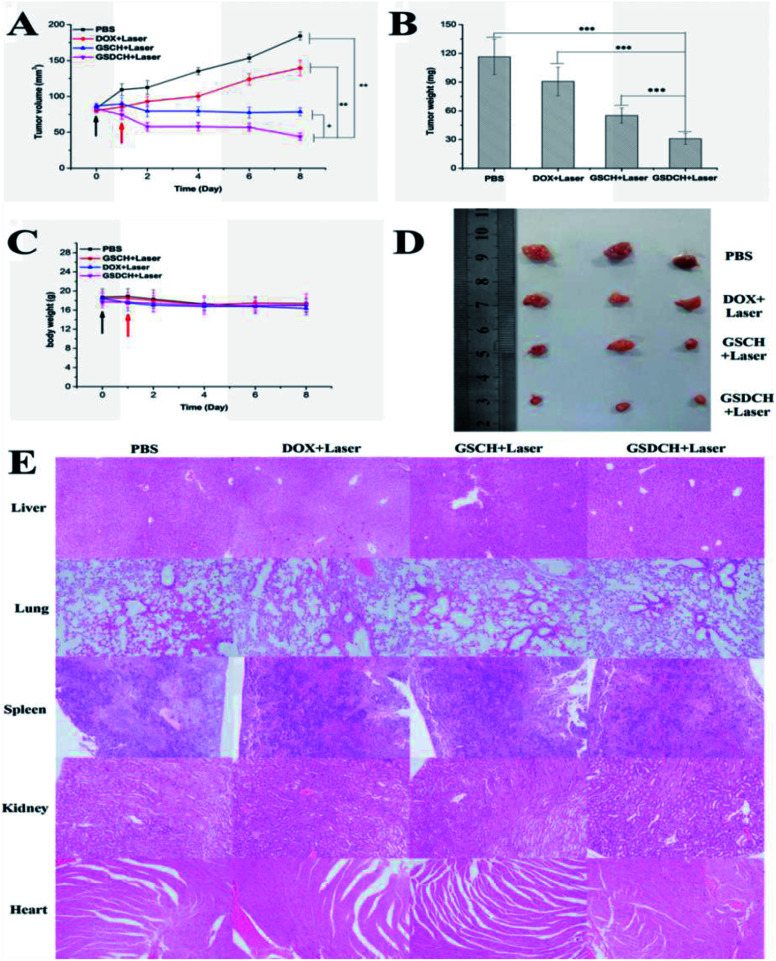
Antitumor study *in vivo via* intravenous injection. (A) Representative photos of mice bearing HeLa tumors at certain points in time. (B) Relative tumor volume and (C) body weight of different groups during treatment with PBS, DOX + laser, GSCH + laser, GSDCH + laser. (D) Photos of excised tumors after treatments. (E) H&E staining images of the major organs of the mice after different treatments. The magnifications were 100×.The data are represented as a mean ± SD (*n* = 7–3). The mice were treated with tail vein injection on day 0 (black arrow) and irradiation on day 1 (red arrow). + no significant difference (*p* < 0.05), **p < 0.01, and ***p < 0.001 were determined by a Student's *t*-test when the group was compared with the groups that were treated with PBS, DOX + laser, GSCH + laser, respectively.

The PTT treatment was conducted 24 h after intravenous injection in nude mice bearing HeLa tumors. The irradiation was performed with 808 nm laser at 2 W cm^−2^ for 5 min. The therapeutic effects were assessed by monitoring the change in tumor volume (Fig. 8A) and tumor weight of mice (Fig. 8B). After treatment finished, body weights (Fig. 8C) and tumor images (Fig. 8D) were investigated. Tumor size monitoring revealed that all tumors in the PBS (control) group grew rapidly. Both the tumor weight and tumor size from the GSDCH + laser treatment group were the lowest among the four groups. Together, these results fully demonstrate that the validity of an integration of chemo-photothermal therapy *in vitro*.

To evaluate the safety concern of GSDCH *in vivo*, H&E staining of those major organs obtained 8 days after irradiation (Fig. 8E) revealed that almost no obvious necrosis occurred in the four treatment groups. The results indicate combination of PTT and chemotherapy by GSDCH might be safe in the present study.

## Experimental section

### Materials

Chloroauric acid (HAuCl_4_·3H_2_O), cetyltrimethylammonium bromide (CTAB) and tetraethoxysilane (TEOS) were purchased from Aladdin (Shanghai China). Ascorbic acid (AA), 5-bromosalicylic acid (5-BrSA) and sodium borohydride (NaBH_4_), silver nitrate (AgNO_3_), hydrochloric acid (HCl), sodium hydroxide (NaOH), doxorubicin hydrochloride (DOX), 3-(4,5-dimethylthiazol-2-yl)-2,5-diphenyltetrazolium bromide (MTT), dimethyl sulfoxide (DMSO) were obtained from Sinopharm Chemical Reagent Co., Ltd. (Shanghai, China).

### Measurement

Ultraviolet-visible-near infrared (UV-vis-NIR) spectra were measured with UV1800 (SHIMADZU). The samples for UV-vis-NIR spectra measurements were prepared by dilution of 200 μL stoste to 2 mL solution. Transmission electron microscopy (TEM) images were measured by Tecnai G2 S-TWIN (FEI). The samples for TEM measurements were prepared by placing 1 mL of sample on copper grids covered with carbon. The samples were observed under TEM after drying. Zeta-potential was measured by a Zetasizer Nano-ZS (Malvern). All measurements were performed in triplicate.

### Cell culture

The HeLa cell line and MCF-7 cell line was bought from the Chinese Academy of Sciences, and maintained in DMEM medium supplemented with 1% penicillin–streptomycin and 10% FBS. The cells were incubated in a cell incubator under 5% CO_2_ atmosphere at 37 °C.

The DMEM medium, FBS, penicillin–streptomycin, trypsin–EDTA and trypsin solutions were purchased from HyClone Corporation (New York, NY).

### Synthesis of CTAB-stabilized GNRs

Gold nanorods (GNRs) were synthesized according to a seed-mediated growth method. The gold solution was prepared first by a reduction reaction, following a fixed routine: 0.5 mL of 0.2 M CTAB aqueous solution was mixed with 0.103 mL of 0.1% (w/v) HAuCl_4_·3H_2_O aqueous solution. 0.1 mL of ice-cold 0.023% (w/v) NaBH_4_ aqueous solution was then quickly added to the mixed solution under vigorous stirring conditions. After that, the solution was kept at 30 °C in a water bath for 30 min before use.

To prepare the growth solution, 1.80 g of CTAB and 0.22 g of 5-BrSA were added to 95 mL of ultrapure water at 60 °C, 2.7 mL of 0.004 M AgNO_3_ and 1 mL of 1 M HCl were then added to the mixed solution. When the solution was cooled to 30 °C, 2.06 mL of 1% (w/v) HAuCl_4_ was added. The solution was kept at 30 °C for 15 min. After that, 0.4 mL of 0.064 M AA was added into the solution and stirring intensely until the solution formed colorless. For GNR growth, 0.08 mL of seed solution was added into the growth solution and the mixed solution was then left undisturbed at 30 °C in water bath for 12 h to the complete growth of nanorods. The enough reacted solution was centrifuged at 10 000 rpm for 30 min and then redispersed in 25 mL of ultrapure water. Different aspect ratios of GNRs were prepared by the change of concentration of AgNO_3_ and HCl.

### Synthesis of mesoporous silica-coated GNRs (GS)

Mesoporous silica-coated GNRs (termed as GS) were synthesized according to a modified Stöber method. Moreover, the thicknesses of mesoporous silica shells were found to be about 30 nm. First, 0.1 mL of NaOH aqueous solution (1 M) was added into 10 mL of as-synthesized GNR solution to adjust the PH value to about 10.0. Then, 2 mL of TEOS-ethanol solution contained 0.1 mL of TEOS was added in the 40 °C of stirring solution batches, the speed of addition was 0.2 mL per 30 min. After continuously stirred at 40 °C for 24 h, the mixture was gathered by centrifugation at 10 000 rpm for 30 min and was repeatedly washed with 10% (v/v) HCl-ethanol solution for six times. The obtained GS were redispersed in 10 mL of ultrapure water.

### Synthesis of carboxyl-modified GNR@SiO_2_ (GSC)

For improvement of encapsulation efficiency of GNR@mSiO_2_@DOX, the functional groups of mesoporous silica-coated shells were modified to be carboxyl groups (termed as GSC). 10 mL of the as-synthesized GNR@SiO_2_ aqueous solution were centrifuged at 10 000 rpm for 30 min and then redispersed in 10 mL of ethanol. 0.1 mL NaOH (1 M) was then added to adjust the pH value to about 10.0 and 2 mL of 0.25% (w/v) APS-COOH aqueous solution was subsequently added. After stirred continuously at room temperature for 12 h, the obtained GNR@SiO_2_–COOH was collected by centrifugation at 10 000 rpm for 30 min and was washed three times with water and ethanol. The products were redispersed in 10 mL of ultrapure water.

### Encapsulation of DOX·HCl

2.5 mg of DOX was dissolved in 2 mL of ultrapure water at a concentration of 1 mg mL^−1^ followed by the addition of 10 mL of GNR@SiO_2_–COOH aqueous solution. The mixture was stirred continuously in a dark environment at room temperature for 48 h. The resultant GNR@SiO_2_-DOX (termed as GSD) was then centrifugation at 10 000 rpm for 30 min and washed with water until the dispersion with no color. The GSD was then redispersed in 10 mL of ultrapure water.

### Synthesis of 7-(double dodecylamine)-4-hydroxymethylcoumarin

The synthesis procedure of 7-(double dodecylamine)-4-hydroxymethylcoumarin was illustrated in ESI.†

### Preparation of GNR@SiO_2_-DOX@CouC12 (GSDC)

100 mg of 1-ethyl-3-(3-dimethylaminopropyl)carbodiimide (EDC) was added into the 10 mL of the as-synthesized GNR@SiO_2_-DOX aqueous solution. After 15 min, 30 mg of 4-dimethylaminopyridine (DMAP) and 30 mg pluronic F-127 were added. After stirred 10 min, 2 mL of 1.5% (w/v) alkylation-hydroxymethyl-coumarin (CouC_12_) trichloromethane solution was added. The specific synthesis procedure of 7-(double dodecylamine)-4-hydroxymethylcoumarin was given in the ESI.† The solution was stirred at room temperature for 12 h and then centrifuged at 10 000 rpm for 30 min and was washed twice with water. The obtained GNR@SiO_2_-DOX@CouC_12_ (termed as GSDC) was kept in 10 mL of ultrapure water.

### Preparation of GNR@SiO_2_-DOX@CouC12-HA (GSDCH)

10 mg of hyaluronic acid (HA) was added into 50 mL of ultrapure water. The mixture was added to 10 mL of the as-synthesized GSDC aqueous solution under vigorous stirring condition. After 12 h, the GNR@SiO_2_-DOX@CouC12-HA (termed as GSDCH) were obtained by centrifuged at 10 000 rpm for 30 min and was washed three times with water and then were kept in 10 mL of ultrapure water for future use.

### Photothermal experiment

1 mL of as-synthesized GNR, GS and GSCH samples dispersed in water by ultrasonication were placed in 2 mL centrifuge tube, irradiated by an adjustable 808 nm laser generator (Changchun new industry photoelectric technology co. LTD, China) for 10 min. The power density was calculated based on the power and the light spot size. The temperature of the aqueous dispersions were measured by a digital thermometer inserted into the aqueous dispersion every 30 s. Pure water was irradiated by the same NIR laser, the temperature change of which was also recorded as control.

### *In vitro* cytotoxicity of GSDCH

*In vitro* cytotoxicities of GSDCH against HeLa and MCF-7 cells were evaluated by MTT viability assay. Cells were cultured with GSDCH at different concentrations from 5 mg mL^−1^ to 200 mg mL^−1^ in the 96-well plate. After incubation for 24 h, 20 μL of 5 mg mL^−1^ MTT was added. The DMEM solution was removed after another 4 h incubation. Finally, 100 mL of DMSO solution was added into each well, and the absorption intensity was recorded at 490 nm using a microplate reader (BioTek).

### Photothermal ablation of cancer cells *in vitro*

HeLa and MCF-7 cells cultured for 24 h in 96-well plate, then aqueous dispersion of GSDH and GSDCH with different concentrations from 5 μg mL^−1^ to 200 μg mL^−1^ were added. After incubation for 24 h, the wells were exposed to irradiation of 808 nm laser (3 W cm^−2^) for 30 s. Then, the cell inhibition rates were measured using MTT assay.

### Cellular fluorescence imaging

HeLa and MCF-7 cells were seeded at a 24-well plate and incubated for 24 h at 37 °C under 5% CO_2_. Then, the GSDCH dispersion equivalent to 25 μg mL^−1^ were added into the each well. After incubation for different time, the cells were washed with PBS three times, followed by the nuclei staining using DAPI solution (Sijiqing Biological Engineering Materials Co., Ltd., Hangzhou). Finally, the fluorescence imaging was collected on an Olympus FV1000 laser-scanning microscope.

### Live/dead cell staining

HeLa and MCF-7 cells cultured for 24 h in 96-well plate, then aqueous dispersion of GSDCH with different concentrations from 5 μg mL^−1^ to 200 μg mL^−1^ were added. After incubation for 12 h, the wells were exposed to irradiation of 808 nm laser (3 W cm^−2^) for 30 s. Then the cells were washed with PBS three times, followed by the nuclei staining using Fluorescein diacetate (FDA) and propidium Iodide (PI) solution (Sijiqing Biological Engineering Materials Co., Ltd., Hangzhou). Finally, the fluorescence imaging was collected on an Olympus FV1000 laser-scanning microscope.

### *In vivo* evaluation of the chemo-photothermal therapy effect

All animal procedures were performed in accordance with the Guidelines for Care and Use of Laboratory Animals of “the Institutional Animal Care and Use Committee (IACUC) of Food and Drug Safety Evaluation Center (Wuhan, China)” and Experiments were approved by the Animal Ethics Committee of “Food and Drug Safety Evaluation Center (Wuhan, China)”. HeLa cells (5 × 10^6^ cell per mL, dispersed into 0.2 mL PBS) were subcutaneously injected into Balb/c nude mice. The administration was carried out when the volume of the tumor reach about 50 mm^3^. 28 tumor-bearing nude mice were randomly divided to four groups (*n* = 7, each group), containing PBS (I), free DOX + laser (II), GSCH + laser (III), and GSDCH + laser (IV). The injection dosage of GSDCH in III and IV groups are 5 mg kg^−1^. The tumors were irradiated with laser (808 nm, 2 W cm^−2^, 5 min) after injection for 24 h. Meanwhile, the mice in group II and group IV were photographed to acquire real-time thermal images using an infrared camera. After treatment, each mouse was weighted with a balance and the dimension of each tumor was monitored by a caliper every two days. The tumor volume (*V*) was computed as follow:
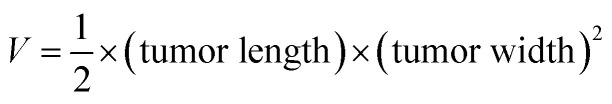


The tumor volume was measured every two days for 8 days.

## Conclusion

In summary, a manually controlled-released multimodal therapy platform GNR@SiO_2_-DOX@CouC12-HA was developed. The DOX was loaded in the mesoporous silica shell and blocked by the hydrophobic coumarin layer. The coumarin layer was disintegrated by NIR light to trigger the DOX release actively. The photothermal efficiency of GSCH was 28.4% under 3 W NIR irradiation. The outside hyaluronic acid also could be selectively accumulated in the HeLa cells. As the integration of photothermal and drug delivery property, the proposed nanocarrier showed superior anticancer efficacy both *in vitro* and *in vivo*. Moreover, due to the present of coumarin layer, NPs could also be used to deliver other drugs, including proteins, DNA, siRNA, *etc.* The results suggest that this manually controlled-release system represents a potential strategy for synergetic chemo-photothermal therapy.

## Conflicts of interest

There are no conflicts to declare.

## Supplementary Material

RA-008-C8RA06176A-s001
