# Supplementary material for: Gold nanorod-based multifunctional nanocarrier for synergistic chemo-photothermal therapy in tumors
Source: RSC Adv. 2018 Dec 11;8(72):41454–63. doi: 10.1039/c8ra06176a (PMC9091941; doi:10.1039/c8ra06176a)
Supplement: RA-008-C8RA06176A-s001 [file RA-008-C8RA06176A-s001.pdf]

Electronic Supplementary Material (ESI) for RSC Advances.
This journal is © The Royal Society of Chemistry 2018

RSC Advances

ARTICLE

Gold Nanorods-based Multifunctional Nanocarrier for Synergistic Chemo-photothermal Therapy in Tumor

Yi Wang#, Qiyao Cui#, Xiaoshuang Zhao, Tang Qin, Wenjing Wang, Hongmei Sun, Hongda Zhu, Huiling Guo, Honghao Sun*

Synthesis of carboxyl-modified 3-aminopropyl triethoxysilane (APTES): 1.9 g of cis-butenedioicanhydride was dissolved in 30 mL of dichloromethane, 9.65 g of 3-aminopropyl)triethoxysilane (APTES) was then dropwise added into the solution in ice water bath. After being stirred 4 h, the products were obtained by evaporated.

Synthesis of m-(N-Methoxycarbonylamino) phenol (MAP): 22 g of m-aminophenol and 25 g of Potassium bicarbonate ($KHCO_3$) was stirred with 150 mL of ethyl acetate and 10 mL water to be suspension. 18 mL of methoxycarbonyl chloride in the constant pressure funnel was then added into the mixture in the ice water bath during 0.5 h. After stirred 1 h, 50 mL of water was added. The mixture was then stirred continuously for 3 h. The aqueous layer was separated, and the organic layer was successively washed with water, 1 M H_2SO_4, water, and saturated NaCl solution, dried with $MgSO_4$, and evaporated. The products were obtained after recrystallization from benzene.

Synthesis of 7-Methoxycarbonylamino-4-methylcoumarin (MAMC): 23 g of MAP and 25 mL of acetoacetic ester was mixed and then added into 60 mL of stirred-concentrated sulphuric acid in batches. After stirred 2 h, the solution was diluted with 300 mL of ice water mixture and then stirred continuously for crystallization of all products. The crystals were dealt with filtration and separation and then sequentially washed with water, methanol and aether. The final products were obtained by desiccation.

Synthesis of 7-amino-4-methylcoumarin (AMC): 28 g of MAMC and 60 mL of 45% (W/V) potassium hydroxide (KOH) aqueous solution were stirred for 20 min at 85 °C. After complete dissolution of MAMC, the solution was reached 200 mL with the desaturation of water. The PH of stirred-solution was carefully adjusted to 6 with hydrochloric acid in ice water bath. The mixture was then alkalized with KOH aqueous solution to PH 8 and stirred for complete separation of crystals. The crystals were washed successively with water, methanol and aether after filtration and separation. The product was dealt with desiccation and collection.

Synthesis of 7-(double dodecylamine)-4- methylcoumarin (DDAMC): 7.98 g of AMC and 33.3 mL of bromododecane were dissolved in 500 mL of N,N-dimethyl formamide (DMF). The solution was then stirred for 20 h at 80 ℃ after the addition of 37.8 g of potassium carbonate (K_2CO_3). The liquid products were obtained after filtration and evaporation of most solvent and then mixed with mass acetone. The mixture was continuously stirred at ice-water bath until complete separation of solid. The products were treated with filtration and then collected.

Synthesis of 7-(double dodecylamine)-4-hydroxymethylcoumarin(DDAHC): 2.0 g of DDAMC and 0.87 g of seleniumoxide were dispersed with 100 mL of dimethylbenzene. The mixture was tempestuously stirred and refluxed for 24 h. The reaction was filtered with siliceousearth and then evaporated for the obtainment of puce oily matter. 0.3 g of sodium metaborate ($NaBH_4$) were added into 70 ml of as-synthesized oily matter methanol solution. After stirred for 4 h, the reaction was then acidized with 1 M HCl aqueous solution to PH 7. The solution was extracted thrice with chloroform. The organic phase was merged and then desiccated with anhydrous magnesium sulfate. The products were obtained after evaporation of solvent.

The photothemal transform efficiency (η) of the materials are described in terms of their absorbed energy (Q). [1,2] For example, the absorbed energy of GNRs (Q_{GNR}), described in terms of the mass of GNRs and GNRs@mSiO$_2$ aqueous dispersion (m_i), the specific heat capacity of water (C_i) and the temperature of their dispersion (T) by

$$\sum_i m_i C_i \frac{dT}{dt} = Q_{GNR} + Q_{sys} - Q_{out} \quad (1)$$

Q_{sys} is the energy supplied for water, Q_{out} is the systematic energy loss.

In theory, the absorbed energy of GNRs (Q_{GNR}) could be calculated by the following equation:

$$Q_{GNR} = I(1-10^{-A_{808}})\eta \quad (2)$$

I is the power of near-infrared laser, A_{808} is the absorbance of GNRs in 808 nm.

Please do not adjust margins

The systematic energy loss (Q_{out}) could be calculated by

$$Q_{out} = hS(T - T_{surr}) \quad (3)$$

h is the thermal transfer coefficient, S is the radiating area, T_{surr} is the ambient temperature. In addition, the energy input and output would achieve a balance when the system temperature reaches a steady state, the equation could be given as:

$$Q_{GNR} + Q_{sys} = Q_{out} = hS(T_{\max} - T_{surr}) \quad (4)$$

When the near-infrared laser is absence, $Q_{GNR} + Q_{sys} = 0$. This results in a modified expression for the system:

$$\sum_i m_i C_i \frac{dT}{dt} = -Q_{out} = -hS(T - T_{surr}) \quad (5)$$

The equation could be change to:

$$dt = -\frac{\sum_i m_i C_i}{hS} \frac{dT}{(T - T_{surr})} \quad (6)$$

A similar expression can be derived:

$$t = -\frac{\sum_i m_i C_i}{hS} \ln \frac{T - T_{surr}}{(T_{\max} - T_{surr})} \quad (7)$$

System time constant (τ_s) is defined as

$$\tau_s = -\frac{\sum_i m_i C_i}{hS} \quad (8)$$

θ is introduced in order to simplify the equation

$$\theta = \frac{T - T_{surr}}{(T_{\max} - T_{surr})} \quad (9)$$

Integrating the equation (8) and equation (9), a new equation is obtained

$$t = -\frac{\sum_i m_i C_i}{hS} \ln \frac{T - T_{surr}}{(T_{\max} - T_{surr})} \quad (10)$$

The system time constant (τs) could also be calculated by cooling time, and the observed energy supplied for water (Q_{sys}) can be expressed by

$$Q_{sys} = hS(T_{\max, H_2O} - T_{surr}) \quad (11)$$

Integrating the above equation

$$Q_{GNR} = I(1 - 10^{-A_{808}})\eta = hS(\Delta T_{GNR} - \Delta T_{H_2O}) \quad (12)$$

$$hS = -\frac{\sum_i m_i C_i}{\tau_s} \quad (13)$$

In addition,

For GNR, with τ_s is equal to 255.7 s, m is 1.0 g and C is 4.2 J/g, hS can be calculated to be 0.0164 W/℃. Substituting *I* = 2.0 W, A_{808} = 0.82, T_{GNR} - T_{H2O} = 36.6 ℃ into Equation. (12), the photothermal conversion efficiency can be determined to be 35.4%.

For GNR@SiO_2, with τ_s is equal to 234.1 s, m is 1.0 g and C is 4.2 J/g, hS can be calculated to be 0.0179 W/℃. Substituting I = 2.0 W, A_{808} = 0.947, $T_{GNR@SiO2}$ - T_{H2O} = 36.5 ℃ into Equation. (12), the photothermal conversion efficiency can be determined to be 36.8%.

For GSCH, with τ_s is equal to 270.0 s, m is 1.0 g and C is 4.2 J/g, hS can be calculated to be 0.0156 W/℃. Substituting I = 2.0 W, A_{808} = 0.953, T_{GSCH} - T_{H2O} = 25.7 ℃ into Equation. (12), the photothermal conversion efficiency can be determined to be 22.6%.

For GSCH, with τ_s is equal to 263.8 s, m is 1.0 g and C is 4.2 J/g, h_S can be calculated to be 0.0159 W/℃. Substituting I = 3.0 W, A_{808} = 0.953, T_{GSCH} - T_{H2O} = 47.7 ℃ into Equation. (12), the photothermal conversion efficiency can be determined to be 28.4%.

References

1 D. K. Roper, W. Ahn and M. Hoepfner, J Phys Chem C Nanomater Interfaces, 2007, 111, 3636.
2 X. Wang, Y. Ma, H. Chen, X. Wu, H. Qian, X. Yang and Z Zha. Colloids and Surfaces B: Biointerfaces, 2017, 152, 449.

This journal is © The Royal Society of Chemistry 20xx

Please do not adjust margins

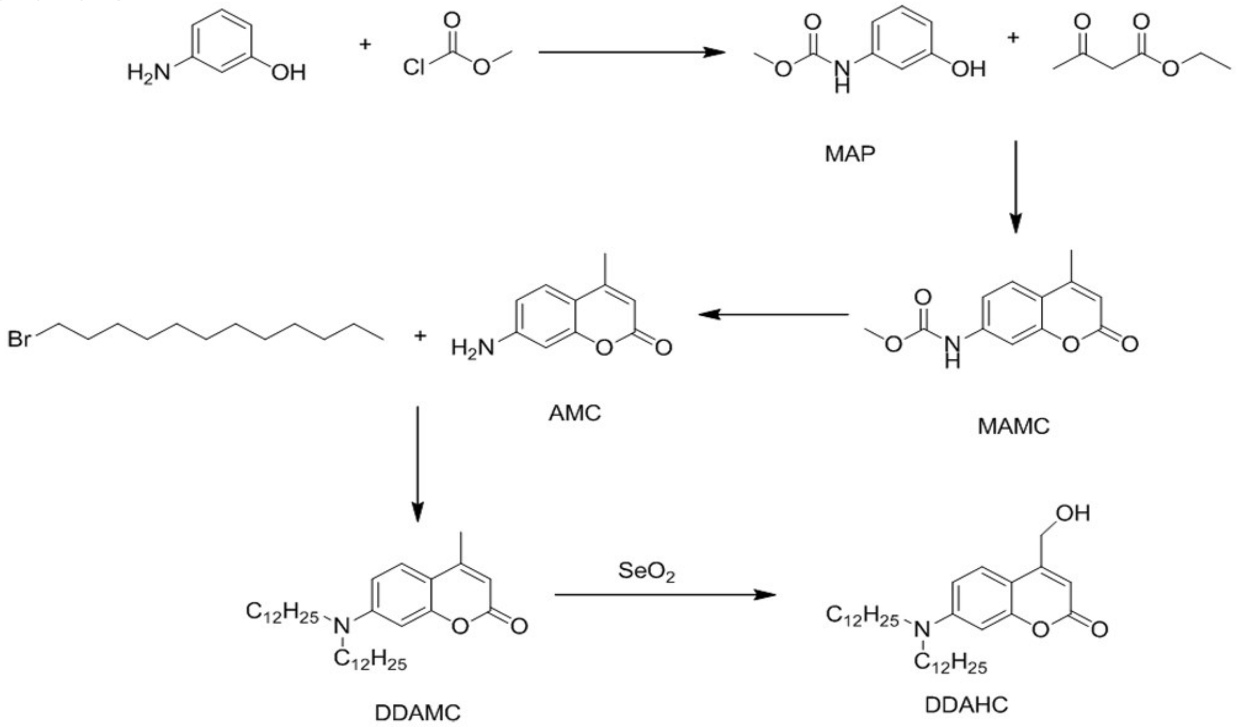

Figure S1. Schematic illustration of the procedure for the synthesis of 7-(double dodecylamine)-4- hydroxymethylcoumarin.

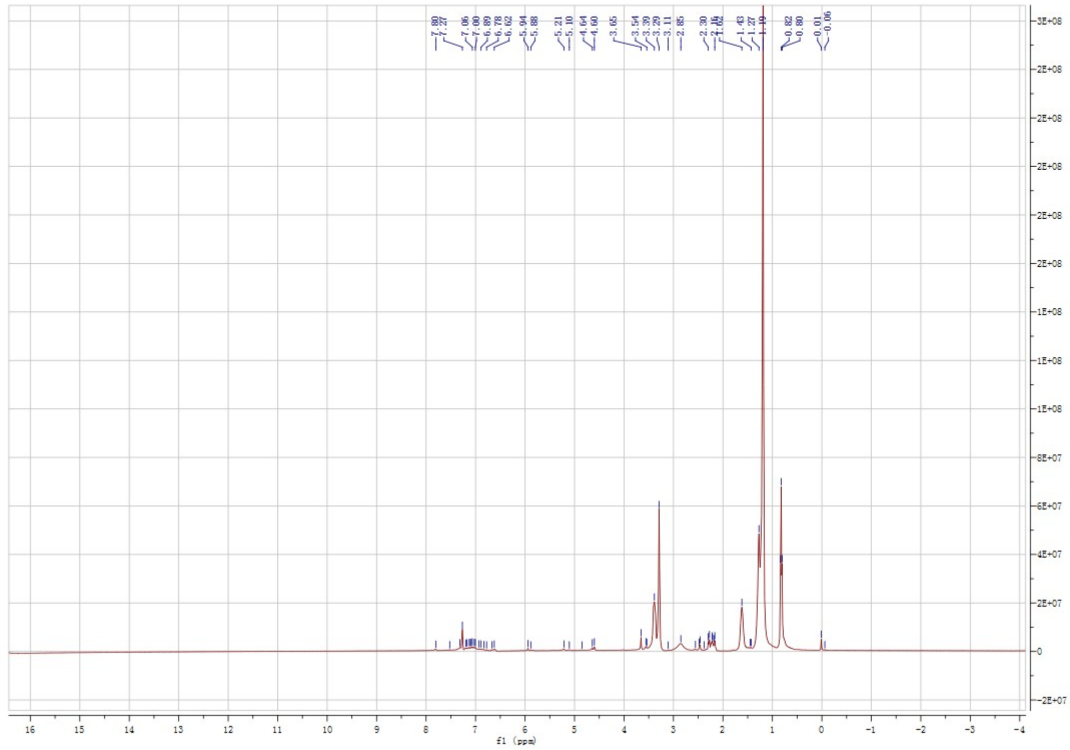

Figure S2. ^1H NMR spectrum of 7-(double dodecylamine)-4-hydroxymethylcoumarin.

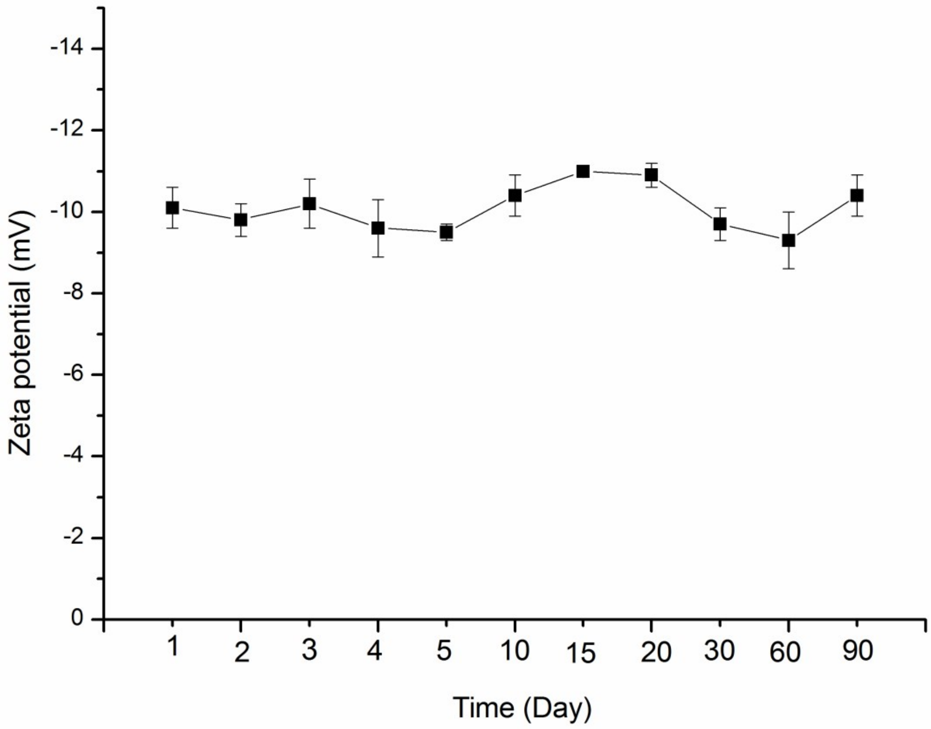

Figure S3. The potential changes of GSDCH over time.

Time(days)	1	2	3	4	5	10	15	20	30	60	90
Size(nm)	106±5	111±4	105±5	121±5	117±5	109±3	115±4	110±5	123±6	120±5	115±6

Table1. The size changes of GSDCH over time.

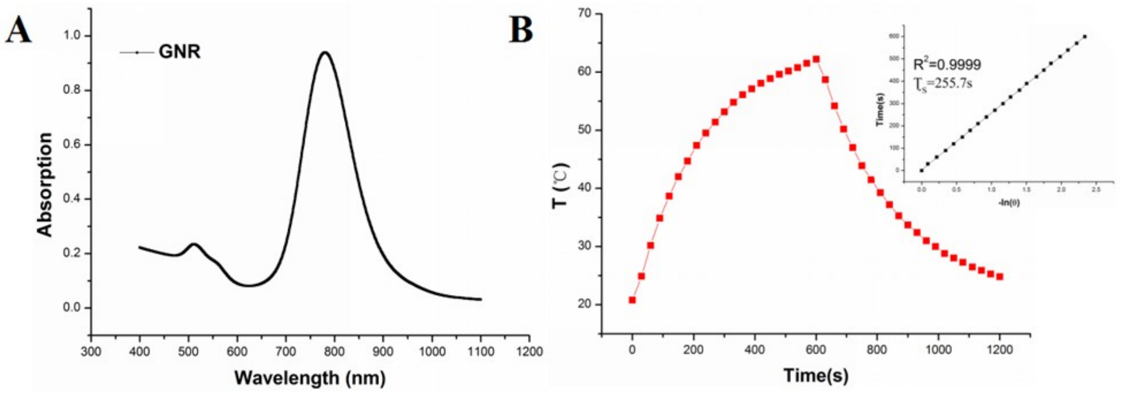

Figure S4. (A) UV-vis-NIR absorption of GNR (1 mg·mL^{-1}), A_{808nm}=0.82. (B) Photothermal effect of the irradiation of the aqueous dispersion of GNR (1 mg·mL^{-1}) with the NIR laser (808 nm, 2 W·cm^{-2}), in which the irradiation lasted to reach the balanceable temperature, and then the laser was shut off.

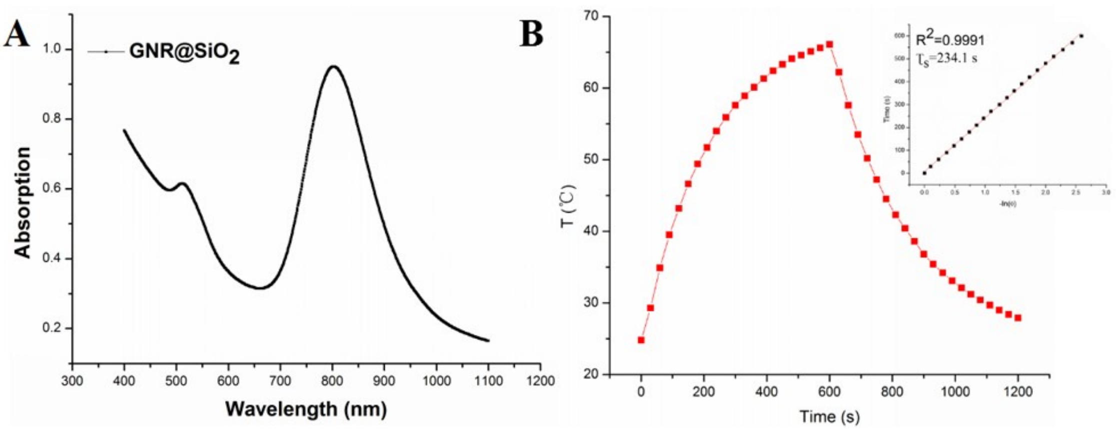

Figure S5. (A) UV-vis-NIR absorption of GNR@SiO_2 (1 mg·mL^{-1}), A_{808nm}=0.947. (B) Photothermal effect of the irradiation of the aqueous dispersion of GNR@SiO_2 (1 mg·mL^{-1}) with the NIR laser (808 nm, 2 W·cm^{-2}), in which the irradiation lasted to reach the balanceable temperature, and then the laser was shut off.

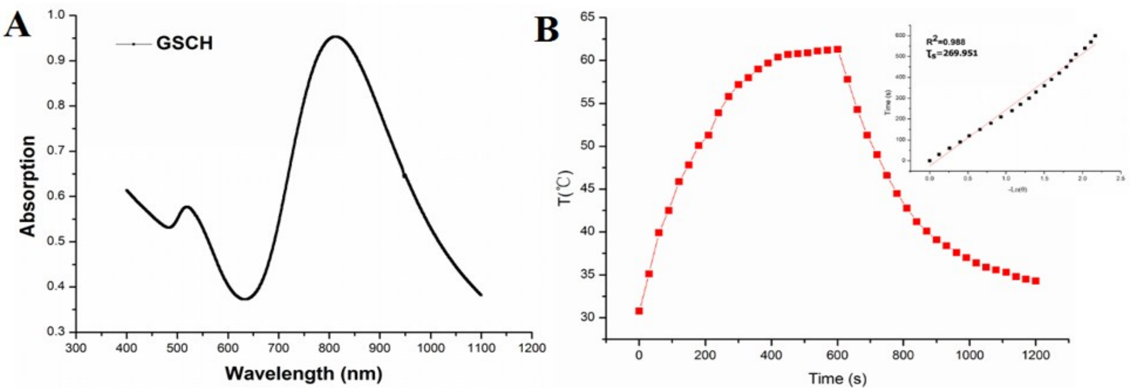

Figure S6. (A) UV-vis-NIR absorption of GSCH (1 mg·mL^{-1}), A_{808nm}=0.953. (B) Photothermal effect of the irradiation of the aqueous dispersion of GSCH (1 mg·mL^{-1}) with the NIR laser (808 nm, 2 W·cm^{-2}), in which the irradiation lasted to reach the balanceable temperature, and then the laser was shut off.

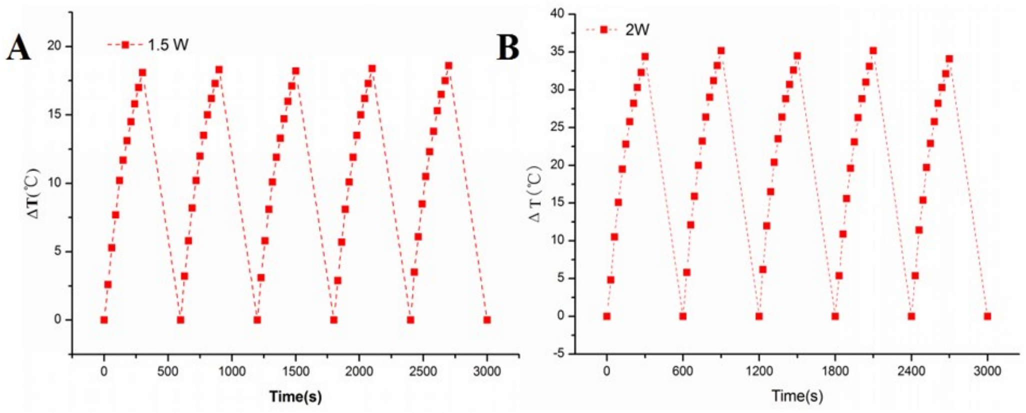

Figure S7. Temperature changes of the aqueous dispersion of GNR (1 mg·mL^{-1}) under repeated on-off irradiation of 808 nm laser at the irradiation power density of (A)1.5 W·cm^{-2} and (B) 2 W·cm^{-2}.

This journal is © The Royal Society of Chemistry 20xx

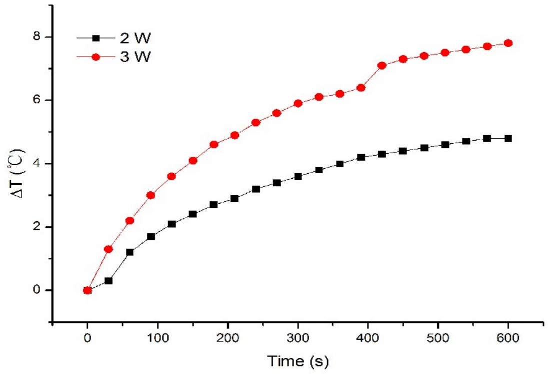

Figure S8. Temperature changes of water with 808 nm laser at the irradiation power density of (A)1.5 W·cm^{-2} and (B) 2 W·cm^{-2}.

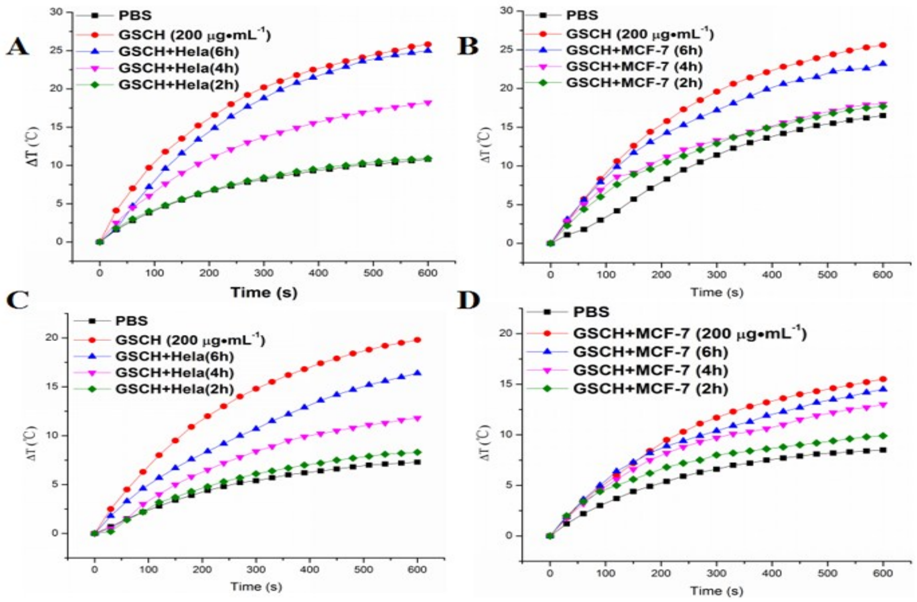

Figure S9. Temperature evolution of GSCH on 808 nm laser irradiation (2 W·cm^{-2} and 3 W·cm^{-2}) for various incubation time with HeLa and MCF-7 cells.

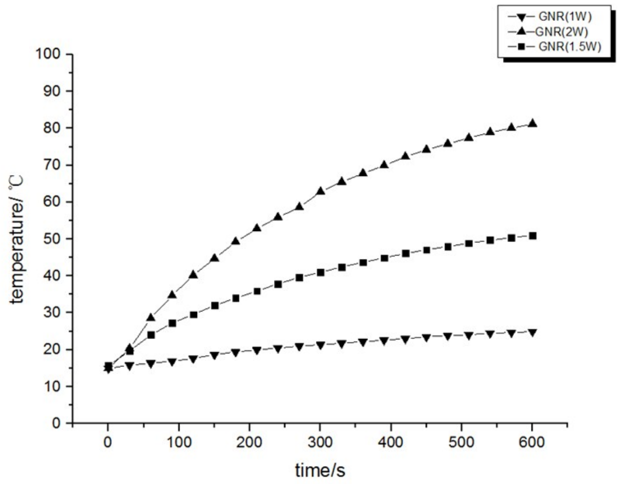

Figure S10. Temperature evolution of GNR in different power densities.

This journal is © The Royal Society of Chemistry 20xx
